# Metabolic response of *Geobacter sulfurreducens *towards electron donor/acceptor variation

**DOI:** 10.1186/1475-2859-9-90

**Published:** 2010-11-22

**Authors:** Tae Hoon Yang, Maddalena V Coppi, Derek R Lovley, Jun Sun

**Affiliations:** 1 Genomatica, Inc., 10520 Wateridge Circle, San Diego, CA 92121, USA; 2 Department of Microbiology, 203 Morrill Science Center IVN, University of Massachusetts, 639 North Pleasant Street, Amherst, MA 01003, USA

## Abstract

**Background:**

*Geobacter sulfurreducens *is capable of coupling the complete oxidation of organic compounds to iron reduction. The metabolic response of *G. sulfurreducens *towards variations in electron donors (acetate, hydrogen) and acceptors (Fe(III), fumarate) was investigated via ^13^C-based metabolic flux analysis. We examined the ^13^C-labeling patterns of proteinogenic amino acids obtained from *G. sulfurreducens *cultured with ^13^C-acetate.

**Results:**

Using ^13^C-based metabolic flux analysis, we observed that donor and acceptor variations gave rise to differences in gluconeogenetic initiation, tricarboxylic acid cycle activity, and amino acid biosynthesis pathways. Culturing *G. sulfurreducens *cells with Fe(III) as the electron acceptor and acetate as the electron donor resulted in pyruvate as the primary carbon source for gluconeogenesis. When fumarate was provided as the electron acceptor and acetate as the electron donor, the flux analysis suggested that fumarate served as both an electron acceptor and, in conjunction with acetate, a carbon source. Growth on fumarate and acetate resulted in the initiation of gluconeogenesis by phosphoenolpyruvate carboxykinase and a slightly elevated flux through the oxidative tricarboxylic acid cycle as compared to growth with Fe(III) as the electron acceptor. In addition, the direction of net flux between acetyl-CoA and pyruvate was reversed during growth on fumarate relative to Fe(III), while growth in the presence of Fe(III) and acetate which provided hydrogen as an electron donor, resulted in decreased flux through the tricarboxylic acid cycle.

**Conclusions:**

We gained detailed insight into the metabolism of *G. sulfurreducens *cells under various electron donor/acceptor conditions using ^13^C-based metabolic flux analysis. Our results can be used for the development of *G. sulfurreducens *as a chassis for a variety of applications including bioremediation and renewable biofuel production.

## Background

*Geobacter *species conserve energy for growth by coupling the complete oxidation of organic compounds to the reduction of Fe(III) and a variety of toxic and radioactive metals [1-4]. *Geobacter sulfurreducens *produce electrically conductive pili that function as nano-wires to promote electron transfer to insoluble electron acceptors such as Fe(III) oxide and electrodes [5,6]. As a result of these properties, *Geobacter *species are utilized to harvest electricity from waste organic matter [7,8] and have served as biocatalysts in microbial fuel cell applications [9,10]. *Geobacter *species have great biotechnological potential for contaminant removal from groundwater and understanding their physiology is crucial for optimizing such applications.

*G. sulfurreducens *has served as a model organism for *Geobacter *species as it is amenable to genetic manipulation and was the first *Geobacter *species to have its genome fully sequenced [11,12]. Constraint-based *in silico *modeling studies have been applied to understand metabolic characteristics of *Geobacter *species [2,4,13]. The *in silico *study of *G. sulfurreducens *metabolism has enabled prediction of the metabolic response of *Geobacter *species to a variety of genetic and culture perturbations in terms of a genome-scale metabolic flux balance analysis. In general, constraint-based *in silico *modeling approaches necessitate a biological objective function such as specific growth rate that can be minimized or maximized in order to predict steady-state metabolic flux distributions. Based upon the overall stoichiometry of the metabolic model, which is typically an underdetermined system, optimal solutions of steady-state fluxes are computed by minimizing/maximizing the selected objective. However, such optimal solutions are objective-dependent, and a proper choice of an objective is non-trivial and can be condition-specific [14]. Therefore, the information predicted by a constraint-based *in silico *approach needs to be interpreted carefully in the context of the actual *in vivo *functional objective of the system.

Metabolic flux analysis using isotopic labeling techniques provide a means to study metabolic pathway activities *in vivo *without the bias of the selection of a particular biological objective function. Amongst available techniques, ^13^C-based metabolic flux analysis (^13^CMFA) has proven to be the most advanced tool for quantifying *in vivo *intracellular pathway activities [15-18]. Modeling and experimental/analytical techniques for ^13^CMFA are well-established, and the method has been utilized to gain a quantitative understanding of a variety of biological systems [15-30]. Recently, ^13^CMFA has also been applied to *Geobacter *to unveil central pathway fluxes in *G. metallireducens *[31] and to elucidate an alternative isoleucine biosynthetic pathway in *G. sulfurreducens *[3].

*G. sulfurreducens *can utilize acetate and hydrogen as electron donors, Fe(III) and fumarate as electron acceptors [32-34] and has a single bifunctional enzyme that catalyzes both fumarate reduction and succinate oxidation [32]. Results from previous results suggest that *G. sulfurreducens *does not utilize fumarate as a carbon source [13,33,35]. However, the intracellular fate of fumarate carbons has not been investigated. The goal of this study was to use ^13^CMFA to quantitatively characterize the *in vivo *intracellular metabolic flux response of *G. sulfurreducens *cultured with different electron donors (acetate and hydrogen) and acceptors (Fe(III) and fumarate). Results from the present work are important for the understanding of the central metabolism of *G. sulfurreducens *and the optimization of bioremediation processes mediated by *Geobacter *species.

## Results and Discussion

### Overview of Experimental Design

We applied ^13^CMFA to characterize the metabolic response of *G. sulfurreducens *to variations in growth conditions. *G. sulfurreducens *was cultured either in chemostats (E1, E3, and E4) or in batch mode (E2) with different combinations of electron donors and acceptors (Table 1). In all experiments, 30% [U-^13^C_2_] acetate was provided as the ^13^C carbon source which also served as the electron donor. In both E1 and E2, Fe(III)citrate served as the electron acceptor. In contrast to E1 where 5 mM acetate served as the electron donor in the chemostat, hydrogen and 1 mM acetate were supplemented under batch culture conditions. In E3 and E4, fumarate was provided as the electron acceptor at different fumarate to acetate ratios to examine the resulting metabolic phenotypes under donor-limiting (E3) and acceptor-limiting (E4) conditions. Selected acetate and fumarate concentrations were based on a previous chemostat study [36] and *in silico *simulations with the *G. sulfurreducens *genome-scale metabolic model which suggested the optimal ratio of fumarate to acetate to be 3.01 for the complete oxidization of acetate [2].

**Table 1:** Cultivation conditions for Geobacter sulfurreducens to study intracellular metabolism with different electron donor/acceptor conditions.

ID	E1	E2	E3	E4
**cultivation mode**	chemostat (*D *= 0.04 h^-1^)	batch	chemostat (*D *= 0.05 h^-1^)	chemostat (*D *= 0.05 h^-1^)
***e*-donor**	5 mM acetate	1 mM acetate & 10 mL H_2_*	5 mM acetate	10 mM acetate
***e*-acceptor**	55 mM Fe(III)citrate	55 mM Fe(III)citrate	28 mM fumarate	20 mM fumarate

*: H_2 _was provided in the headspace of the culture (initial partial pressure ~ 0.60 atm).

### Steady-State Effluxes in Chemostat Cultures

Extracellular yield coefficients for the three sets of chemostat cultures (E1, E3, E4) were determined from steady-state rate measurements. The complete oxidation of acetate to CO_2 _releases of 8 electrons, which is sufficient to convert 8 moles of Fe(III) to Fe(II) or 4 moles of fumarate to succinate. For E1 (5 mM acetate + 55 mM Fe(III)citrate), the molar yield of Fe(II) per mole of acetate consumed was 7.1 ± 0.5, suggesting that only 11.3% of the acetate in the growth medium was utilized for biomass synthesis. When fumarate was used as the electron acceptor, the molar yield of succinate per mole of acetate consumed was 2.9 ± 0.2 for E3 (5 mM acetate + 28 mM fumarate) and 2.5 ± 0.2 for E4 (10 mM acetate + 20 mM fumarate), indicating that a higher percentage of acetate, 27.5% to 37.5%, was utilized for biomass synthesis. The molar yield of fumarate uptake on acetate was 2.8 ± 0.1 and 2.5 ± 0.1 in E3 and E4, respectively. The biomass yield coefficient on acetate (Y_X/DON = _gram dry cell weight produced per mmol acetate uptake) of the Fe(III)citrate culture (E1) was 2.8 × 10^-3 ^± 2 × 10^-4 ^g/mmol, about 4-fold lower than those of the fumarate cultures, 0.013 ± 2 × 10^-3 ^and 0.012 ± 2 × 10^-3 ^for E3 and E4, respectively. Extracellular concentration measurements can be associated with larger uncertainties than mass spectrometric carbon labeling measurements. Thus, the effluxes were set as free parameters to be estimated from ^13^C labeling measurements during ^13^CMFA or calculated later from *in vivo *flux estimates.

### Metabolic Fate of Citrate & Fumarate Carbons

In order to assess metabolic flux distributions in *G. sulfurreducens *grown with Fe(III)citrate as the electron acceptor, we needed to determine whether *G. sulfurreducens *is able to metabolize citrate in addition to acetate. We also investigated whether *G. sulfurreducens *can utilize fumarate as a carbon source since previous studies have suggested that fumarate is not utilized as a carbon source [13,33,35,37]. To this end, cells were grown in a batch culture containing 10 mM non-labeled acetate with 55 mM Fe(III)citrate (4.2% [U-^13^C_6_]Fe(III)citrate) and 10 mM non-labeled acetate with 27.5 mM fumarate (30% [2,3-^13^C_2_]fumarate), respectively. The carbon mass isotopomer distributions (MID_AA_) of alanine, glycine, valine, leucine, isoleucine, phenylalanine, serine, aspartate, and glutamate were compared to those obtained from an identical culture containing only non-labeled compounds.

Linear regression of the two sets of MID_AA _measured in the acetate/Fe(III)citrate culture gave a slope of 1.003 with *R*^2 ^of 1.000 and an intercept of 0.000. In addition, the root mean square (RMS) [38] of the difference between the two MID_AA _sets was 0.0006, which was significantly less than the average MID_AA _determined measurement error (ca. 0.003) [27]. This result indicates that the citrate carbons were not utilized by *G. sulfurreducens *cells, Fe(III)citrate served exclusively as an electron acceptor, and that acetate was the sole carbon source in experiments E1 and E2.

In contrast, the acetate/fumarate culture gave a distinct difference from the natural abundance (slope = 1.151, intercept = 0.030, *R*^2 ^= 0.9326) with an RMS of 0.011. For instance, MID_AA _of aspartate gave the largest discrepancy from the natural abundance (RMS = 0.08), followed by glycine (RMS = 0.054), glutamate (RMS = 0.049), serine (RMS = 0.044), phenylalanine (RMS = 0.032), isoleucine (RMS = 0.012), alanine, leucine, and valine (RMS = 0.004). Our results demonstrate that fumarate carbons can be metabolized into different amino acid biosynthesis precursors in the central metabolic pathways. Cellular fumarate utilization was investigated more quantitatively in terms of ^13^CMFA.

### Assessment of Biological Reproducibility

The consistency between biological replicates was evaluated by comparing the MID_AA _of replicates. The squared correlation coefficients (*R*^2^) between the MID_AA _values of the biological replicates (*n *= 3) were higher than 0.9990 in all the chemostat experiments (E1, E3, E4), suggesting a high consistency between the replicates. Thus, the experimental data were considered to be unbiased by biological variations, and the intracellular metabolic fluxes were computed using the mean values of replicate MID_AA _and measured effluxes.

### Comparison of Amino Acid Labeling Patterns

**Figure 1:**
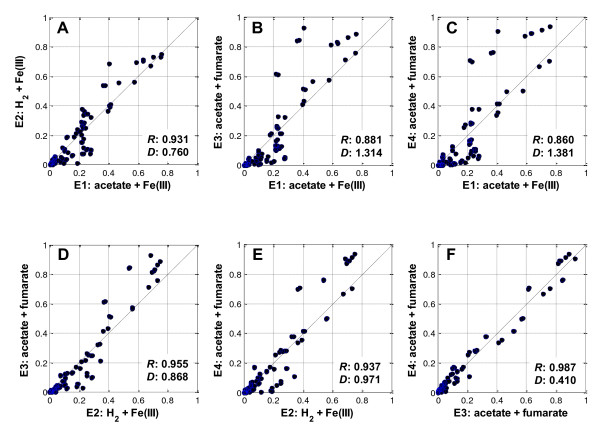
**Comparisons of amino acid carbon mass isotopomer distributions between the experiments listed in Table 1**. The straight line corresponds to a one-to-one (y = x) line. ***R***: correlation coefficient; ***D***: Euclidian distance.

^13^C-labeling patterns of metabolic products including proteinogenic amino acids are not direct measures of the in vivo flux distributions, but information on the flux distributions is recorded in the labeling patterns. Therefore, gross metabolic discrepancies or similarities induced by the different electron donor/acceptor conditions can be visualized by directly comparing the MID_AA _values obtained from these conditions. As shown in Figure 1, the comparison of MID_AA _values suggested that electron acceptor variation (Figure 1-B, C, D, E: Fe(III) vs. fumarate) resulted in a larger discrepancy in ^13^C labeling patterns than electron donor variation (Figure 1-A: acetate vs. hydrogen). In terms of the Euclidian distance, the measure of the difference of two sets of data; the MID_AA _values from E3 to E1 (Figure 1-B) or E4 to E1 (Figure 1-C) was larger than that from E2 to E1 (Figure 1-A). The distance from E2 to E3 or E4 (Figure 1-D, E) was smaller than that from E1 to E3 or E4, however larger than the distance between E1 and E2. The most similar amino acid ^13^C labeling patterns were found between E3 and E4, in which only donor and acceptor concentrations were varied (Figure 1-F). Nevertheless, detectable differences of MID_AA _between E3 and E4 indicate that the physiological state of *G. sulfurreducens *is altered in response to varying donor and acceptor concentrations.

### Overview of in vivo Fluxes

**Figure 2:**
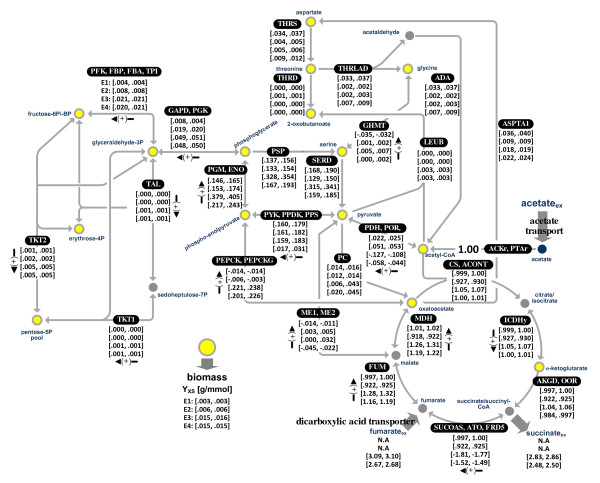
***In vivo *flux estimates (95% confidence intervals given for the flux mean estimates) for the central metabolic network of *G. sulfurreducens*, corresponding to E1, E2, E3, and E4 (Table 1)**. All fluxes are normalized to acetate uptake (ACKr, PTAr). Gray double-headed arrows indicate bidirectional reactions, and the black arrowa with plus signs correspond to the net direction of a positive value for the bidirectional reactions. Unidirectional reactions were indicated by gray single-headed arrows. The subscript "*ex*" denotes extracellular metabolites. The abbreviations (pathway enzymes) are listed in Table 2, and precursor demand for biomass synthesis (precursors in yellow color) in Table 4.

To assess *in vivo *flux distributions from the four cultures of *G. sulfurreducens*, 500 stochastic numerical optimization runs were performed by generating random numbers of measurements (from the normal distributions estimated using experimentally determined means and standard deviations) and starting points (from a continuous uniform distribution between the upper and lower bound of each parameter). The details enzyme reactions comprised in the metabolic network are listed in Table 2, and the 95% confidence intervals for the resulting flux estimates are depicted in Figure 2. The fluxes were normalized to acetate uptake and can be understood as molar yield coefficients on acetate. For each experiment, the experimental measurements and the corresponding model-predicted values gave a high degree of consistency; their correlation coefficients (*R*) were higher than 0.9970 at the optimum with the slope and *y*-axis intercept close to 1 and 0, respectively.

**Table 2:** Abbreviations and EC numbers of the enzymes considered in the central metabolic network of G. sulfurreducens for 13C metabolic flux analysis.

abbreviation	EC number	enzyme	
**ACKr**	2.7.2.1	acetate kinase	
**PTAr**	2.3.1.8	phosphotransacetylase	
**PFK**	2.7.1.11	phosphofructokinase	
**FBP**	3.1.3.11	fructose-bisphosphatase	
**FBA**	4.1.2.13	fructose-bisphosphate aldolase	
**TPI**	5.3.1.1	triose-phosphate isomerase	
**GAPD**	1.2.1.12	glyceraldehyde-3-phosphate dehydrogenase (NAD)	
**PGK**	2.7.2.3	phosphoglycerate kinase	
**PGM**	5.4.2.1	phosphoglycerate mutase	
**ENO**	4.2.1.11	enolase	
**PYK**	2.7.1.40	pyruvate kinase	
**PPDK**	2.7.9.1	pyruvate phosphate dikinase	
**PPS**	2.7.9.2	phosphoenolpyruvate synthase	
**PDH**	1.2.1.51	pyruvate dehydrogenase	
**POR**	1.2.7.1	pyruvate synthase (pyruvate ferredoxin oxidoreductase)	
**TKT1**	2.2.1.1	transketolase	
**TAL**	2.2.1.2	transaldolase	
**TKT2**	2.2.1.1	transketolase	
**CS**	4.1.3.7	citrate synthase	
**ACONT**	4.2.1.3	aconitase	
**ICDHy**	1.1.1.42	isocitrate dehydrogenase (NADP)	
**AKGD**	1.2.4.2	2-oxoglutarate dehydrogenase	
**OOR**	1.2.7.3	2-oxoglutarate synthase	
**SUCOAS**	6.2.1.5	succinyl-CoA synthetase (ADP-forming)	
**ATO**	2.8.3.8	acetate CoA-transferase	
**FRD5**	1.3.5.1	succinate dehydrogenase (menaquinone 7)	
**FUM**	4.2.1.2	fumarase	
**MDH**	1.1.1.37	malate dehydrogenase	
**PC**	6.4.1.1	pyruvate carboxylase	
**ME1**	1.1.1.38	malic enzyme (NAD)	
**ME2**	1.1.1.40	malic enzyme (NADP)	
**PEPCK**	4.1.1.49	phosphoenolpyruvate carboxykinase	
**PEPCKG**	4.1.1.32	phosphoenolpyruvate carboxykinase (GTP)	
**ASPTA1**	2.6.1.1	aspartate transaminase	
**THRS**	4.2.3.1	threonine synthase	
**THRD**	4.3.1.19	threonine deaminase	
**LEUB**	1.1.1.85	3-isopropylmalate dehydrogenase	
**THRLAD**	4.1.2.5	threonine aldolase	
**ADA**	1.2.1.10	acetaldehyde dehydrogenase	
**GHMT**	2.1.2.1	glycine hydroxymethyltransferase	
**PSP**	3.1.3.3	phosphoserine phosphatase	
**SERD**	4.3.1.17	serine deaminase	

Under the examined four conditions, the majority of consumed acetate was oxidized through the TCA cycle (Figure 2). When comparing fluxes in E1 and E2, the flux through the initial reaction of the TCA cycle (CS/ACONT) in the H_2_/Fe(III) culture with 1 mM acetate (E2) was significantly less than that obtained from the acetate/Fe(III) culture (E1). The addition of hydrogen increased the flux from acetyl-CoA to pyruvate (POR: pyruvate synthase) as well as other gluconeogenetic fluxes and resulted in a 2-fold higher biomass yield on acetate (*cf*. Y_XS _values in Figure 2). The coupling of Fe(III) reduction to H_2 _oxidation, an energy generating process resulted in decreased demand for energy generation by the oxidation of acetyl-CoA via the TCA cycle and therefore increased the fluxes of acetate carbons for gluconeogenesis, biomass synthesis, and other anabolic activities.

Surprisingly, when fumarate was provided as the electron acceptor in both E3 and E4, flux through the initial reactions of the TCA cycle (CS/ACONT) was slightly higher than 1, suggesting that acetate was not the sole source of carbon for the TCA cycle. In addition to acetate, fumarate served as carbon source in these cultures and likely introduced the additional carbons into the TCA cycle. The *in vivo *flux distribution suggests that when fumarate was provided as the electron acceptor, fumarate was not only reduced to succinate but also converted to malate by fumarase (FUM) and further to oxaloacetate via malate dehydrogenase (MDH). During cell growth with fumarate as the electron acceptor, synthesis of oxaloacetate from fumarate contributed significantly to gluconeogenesis via its conversion to phosphoenolpyruvate by phosphoenolpyruvate carboxykinase (PEPCK/PEPCKG in Figure 2). In addition, there was a significant net conversion of phosphoenolpyruvate into acetyl-CoA (via the combined actions of PDH and/or POR) in the fumarate cultures. This flux accounted for the elevation in the TCA cycle relative to the acetate uptake flux. The fumarate uptake was slightly higher than the succinate secretion, indicating the consumption of fumarate. Our ^13^CFMA results suggest that fumarate serves not only as an electron acceptor, but also as a carbon source and to a lesser extent, as an electron donor. This finding contrasts with those of previous cultivation and genetic studies [13,33,35,37] which suggested that fumarate was not utilized as a carbon source or an electron donor.

In contrast to the gluconeogenesis during growth with fumarate, no significant gluconeogenetic flux through PEPCK/PEPCKG was observed during growth with Fe(III) citrate, where the conversion of acetyl-CoA into pyruvate by PDH/POR was the main gluconeogenetic flux (Figure 2). In all cases, a small flux through PC was observed that seemed to be necessary for anaplerosis towards oxaloacetate to initiate the TCA cycle. As stated above, PEPCK/PEPCKG also showed anaplerotic function including ATP production and carboxylation in both Fe(III) cultures (E1 and E2), although its contribution to the anaplerosis in the culture with hydrogen (E2) was insignificant.

The flux distributions in acetate/fumarate chemostats E3 and E4 revealed that growing *G. sulfurreducens *under donor-limiting conditions (E3) versus acceptor-limiting conditions (E4) resulted in differences in fumarate uptake and succinate secretion effluxes, as well as in the utilization of the fumarate carbon source. In both cases, the net fumarate uptake flux was higher than the sum of succinate secretion, further suggesting that fumarate was utilized as an additional carbon source. The reductive flux of SUCOAS/ATO/FRD5 was higher in the donor-limited chemostat than that in the acceptor-limited conditions. Fumarate uptake and succinate secretion effluxes were also larger in E3 than in E4. In addition, the oxidative TCA fluxes (CS/ACONT, AKGD/OOR, FUM, MDH) in E3 were slightly higher than those in E4. Our results suggest that excessive fumarate in E3 resulted in elevation of the reductive flux of succinate dehydrogenase in conjunction with increased oxidative TCA fluxes. Furthermore, excessive fumarate present in E3 elevated the initial flux of gluconeogenesis carried out by PEPCK/PEPCKG. A more detailed investigation of the fumarate cultures is provided in a later section entitled '*Fumarate Utilization*'.

### Branch Point Flux Distributions

**Figure 3:**
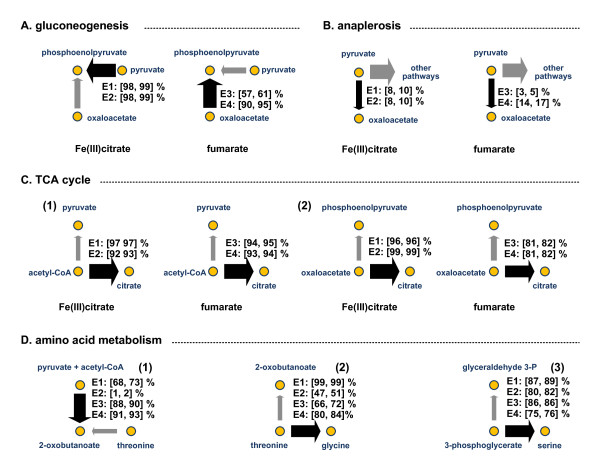
**Relative flux distributions (95% confidence interval) at key branch points of the central metabolic network for different experimental conditions of E1, E2, E3, and E4 (Table 1)**.

Based on a one-way ANOVA conducted at a significance level of 0.05, none of the relative flux estimates were equal in all four experiments, with the exception of the anaplerotic flux of PC. Likewise, comparison of relative fluxes for all possible pair-wise distributions revealed only a few instances in which fluxes were identical. Although many of the flux discrepancies between experiments may be attributable to differences in acetate uptake and/or biomass formation, some fluxes were not expected to vary. For instance, the fluxes from phosphoglycerate to glucose 6-phosphate in gluconeogenesis and pentose phosphate pathway fluxes should be proportional to the anabolic demand under all four conditions. To gain detailed understanding of the metabolic response of *G. sulfurreducens *under varying growth conditions, relative flux distributions at key branch points in gluconeogenesis, anaplerosis, the TCA cycle, and amino acid metabolism were examined in detail (Figure 3).

The synthesis of phosphoenolpyruvate is one key branch point of gluconeogenesis. As shown in Figure 3-A, the major pathway for phosphoenolpyruvate synthesis for gluconeogenesis varied with electron acceptors. When Fe(III) was the electron acceptor (E1 & E2), phosphoenolpyruvate for gluconeogenesis was generated almost exclusively (89 - 99%) from pyruvate by PYK/PPDK/PPS, and phosphoenolpyruvate synthesis via the decarboxylation of oxaloacetate (PEPCK/PEPCKG) did not contribute significantly to gluconeogenesis. In contrast, when fumarate was provided as the electron acceptor (E3 & E4), the decarboxylation of oxaloacetate (PEPCK/PEPCKG) was the main route for phosphoenolpyruvate biosynthesis for gluconeogenesis. In the acceptor-limited culture (E4), phosphoenolpyruvate for gluconeogenesis was synthesized almost exclusively from oxaloacetate (ca. 93%), whereas in the donor-limited culture ca. 60% of phosphoenolpyruvate for gluconeogenesis was generated from oxaloacetate by PEPCK/PEPCKG by consuming ATP/GTP.

Conversion of pyruvate to oxaloacetate by pyruvate carboxylase (PC) plays a key role in anaplerosis. As shown in Figure 3-B this branch point of anaplerosis was less sensitive towards electron acceptor variation than gluconeogenesis. In the both Fe(III) cultures, ca. 9% of pyruvate was converted into oxaloacetate for anaplerosis, whereas the amount of pyruvate that was diverted to anaplerosis varied from 4 to 15% during growth with fumarate. This variation appeared to reflect the degree to which oxaloacetate was diverted to gluconeogenesis. In the acceptor-limited culture (E4), where 90-95% of the oxaloacetate synthesized from fumarate was used for gluconeogenesis, about 15% of pyruvate was utilized for anaplerosis. In contrast, in the donor-limited culture (E3), 57-61% of oxaloacetate was consumed for gluconeogenesis and 4% of pyruvate was diverted to anaplerosis.

The condensation of acetyl-CoA and oxaloacetate (CS/ACONT) is the initiation step of the TCA cycle. As shown in Figure 3-C.1, the relative amount of acetyl-CoA (between 92% and 97%) entering the TCA cycle was comparable for all cultivation conditions. The relative amount of acetyl-CoA entering the TCA cycle was nearly identical for the two fumarate cultures (E3 and E4). In the Fe(III) cultures, the percentage of acetyl-CoA entering the TCA cycle in the culture with acetate as the sole electron donor (97%) was higher than that in the presence of both hydrogen and acetate as the electron donors (92%). Examination of oxaloacetate utilization (Figure 3-C.2) revealed that nearly all of the oxaloacetate produced during growth via Fe(III) reduction (96 - 99%) was utilized to initiate the TCA cycle, whereas only 82% to 85% of oxaloacetate utilized to initiate the TCA cycle in the two fumarate cases. This difference can be attributed to the utilization of fumarate as an additional carbon source for gluconeogenesis and amino acid biosynthesis.

A recent study [3] indicated that *G. sulfurreducens *has two pathways for isoleucine biosynthesis: the precursor 2-oxobutanoate can be generated (*i*) by the deamination of threonine and (*ii*) from pyruvate and acetyl-CoA through the citramalate pathway. Risso et al. (2008) [3] observed that the citramalate pathway accounted for the majority of isoleucine biosynthesis during growth with fumarate. This was also the case in the present study where 88 to 93% of 2-oxobutanoate was synthesized from pyruvate and acetyl-CoA in the two fumarate cultures (Figure 3-D.1). A smaller fraction of 2-oxobutanoate, ca. 70%, was produced via the citramalate pathway in the acetate Fe(III) culture (E1). In contrast, the 2-oxobutanoate was synthesized almost exclusively from threonine (98 - 99%) in E2 culture containing hydrogen, Fe(III), and 1 mM acetate. During growth in the presence of hydrogen, decreased demand for energy production by the TCA cycle may result in TCA cycle intermediates such as oxaloacetate (main precursor for aspartate and threonine), more available for other uses such as amino acid biosynthesis. In fact, a complete TCA cycle is not required during growth on hydrogen [13].

*G. sulfurreducens *also has two pathways for the biosynthesis of glycine: through the conversion of threonine to glycine and acetaldehyde by threonine aldolase (THRLAD) and from the conversion of serine to glycine by glycine hydroxymethyltransferase (GHMT). ^13^CMFA indicated that the percentages of threonine converted into glycine and acetaldehyde varied under different conditions (Figure 3-D.2). In E1, 99% of threonine was converted to glycine and acetaldehyde. The acetaldehyde was further converted to acetyl-CoA (generating NADH) and the acetyl-CoA was then recycled into the central metabolic pathways.

Another interesting observation which has not been previously described in the literature was that a large portion of 3-phosphoglycerate (75 - 89%) was converted to serine (Figure 3-D.3). At this metabolic branch point, the flux into glyceraldehyde 3-phosphate for gluconeogenesis was kept at a relatively low level corresponding to anabolic demands. Serine was subsequently converted to pyruvate by serine deaminase. As shown in Figure 2, there were significant fluxes through serine. During growth with Fe(III) as the electron acceptor (E1, E2), there was a ATP-consuming futile cycle consisting of phosphoenolpyruvate, phosphoglycerate, serine, and pyruvate, where phosphoenolpyruvate synthase (PPS) and/or pyruvate phosphate dikinase (PPDK) consume ATP when converting pyruvate to phosphoenolpyruvate.

In spite of its small contribution to 2-oxobutanoate biosynthesis, a high level of threonine deaminase activity (25-higher than that of citramalate synthase) has been previously detected in soluble extracts of *G. sulfurreducens *growing on fumarate [3]. The threonine deaminase of *G. sulfurreducens *is also capable of deaminating serine to pyruvate [3], and is likely the enzyme deaminating serine in *G. sulfurreducens*, as this species does not contain the Fe(II)-dependent and oxygen sensitive serine deaminases gene found in *Clostridia *and plants. Moreover, knocking out the threonine deaminase eliminated all detectable serine deaminase activity in soluble extracts prepared under aerobic conditions [3]. Given the presence of relatively high levels of serine deaminase activity in *G. sulfurreducens *extracts, the relatively high flux through serine deaminase that observed by ^13^CMFA is plausible.

It has been hypothesized that futile cycles in cellular metabolism are involved in the regulation of biochemical pathways. Quian and Beard (2006) [39] suggested that futile cycles actively shift the effective equilibrium by expending energy where the magnitude of changes in effective equilibria and sensitivities is a function of the amount of energy used by a futile cycle. Also, it has been suggested that cellular regulation of the ATP/ADP ratio depends on both the specific growth rate and the environmental conditions [40]. Tang et al. (2007) [31] has found futile cycle activity in *Geobacter metallireducens*, however metabolic function of futile cycles is yet fully understood. The observed futile cycle might be related to an important metabolic function, e.g., in regulating substrate utilization for gluconeogenesis and the oxidative TCA cycle.

### Specific Metabolic Fluxes

In addition to the relative branch point flux distributions, we compared the specific metabolic fluxes, which are quantities scaled by cell growth (see Methods Eq. 4), to understand the growth-associated metabolic activities. As shown in Table 3, the specific fluxes from phosphoglycerate to hexose 6-phosphate of the lower gluconeogenesis and pentose phosphate pathways were invariant under different culture conditions. These results suggest that these pathways operated purely to satisfy anabolic demand (Table 4) and that the differences in the relative fluxes through these pathways shown in Figure 2 were due to different biomass yields on acetate under different conditions. The specific fluxes of the upper gluconeogenesis pathway from pyruvate to phosphoenolpyruvate were similar to those from phosphoenolpyruvate to phosphoglycerate during growth with Fe(III) (E1 & E2). In comparison, these specific fluxes were considerably lower than those from phosphoenolpyruvate to phosphoglycerate when fumarate was the electron acceptor. This appeared to be due to the substantial contribution of the phosphoenolpyruvate carboxykinase to gluconeogenesis by converting oxaloacetate to phosphoenolpyruvate.

**Table 3:** Specific fluxes (fluxes normalized by the biomass yield estimate: (mmolmetablite/h)(gbiomass/h)-1) in the central metabolic pathways of the experiments listed in Table 1.

pathway enzymes	specific metabolic fluxes				pathway description
		
	E1	E2	E3	E4	
ACKr, PTAr	369	166	65.2	66.3	acetate uptake

PFK, FBP, FBA, TPI	1.38	1.38	1.38	1.38	lower gluconeogenesis
GAPD, PGK	3.25	3.25	3.25	3.25	

PGM, ENO	57.5	27.1	25.6	15.3	upper gluconeogenesis
PYK, PPDK, PPS	62.6	28.4	11.1	1.60	

PDH, POR	8.68	8.68	-7.63	-3.37	pyruvate ↔ acetyl-CoA

PEPCK, PEPCKG	-4.62	-0.77	15.0	14.2	gluconeogenesis initiation

TKT1	0.05	0.05	0.05	0.05	pentose phosphate pathway
TAL	0.05	0.05	0.05	0.05	
TKT2	0.31	0.31	0.31	0.31	

CS, ACONT	370	154	69.3	66.5	TCA cycle
ICDHy	370	154	69.3	66.5	
AKGD, OOR	370	153	68.5	65.7	
FRD5	370	153	-116	-99.5	
FUM	370	153	84.8	77.8	
MDH	374	153	83.8	80.0	

decarboxylic acid transporter	/	/	202	177	fumarate uptake
	/	/	185	165	succinate secretion

Enzyme abbreviations are listed in Table 2.

**Table 4:** Precursor demand of G. sulfurreducens from the intermediary metabolites involved in the central metabolic pathways in Figure 2.

Precursor demand of *G. sulfurreducens*	
**precursor**	**demand [mmolg_biomass_^-1^]**

glucose 6-phosphate	0.964
fructose 6-phosphate	0.056
ribose 5-phosphate	0.405
erythrose 4-phosphate	0.259
glyceraldehyde 3-phosphate	0.192
3-phosphoglycerate	0.062
phosphoenolpyruvate	0.519
pyruvate	1.842
acetyl-CoA	3.556
α-ketoglutarate	0.755
aspartate	0.814
serine	0.490
glycine	0.576
threonine	0.173
2-oxobutanoate	0.198

As indicated in Figure 2, the initial step in gluconeogenesis was the generation of pyruvate from acetyl-CoA (POR) in the Fe(III) cultures and phosphoenolpyruvate formation from oxaloacetate by PEPCK/PEPCKG in the fumarate cultures. As shown in Table 3, the specific fluxes of POR were identical in the two Fe(III) cultures, and the specific fluxes of PEPCK/PEPCKG in the fumarate cultures were similar. Thus, the initial gluconeogenetic flux per unit biomass synthesis remained constant when the same electron acceptor was utilized.

Also shown in Table 3, the specific flux through the TCA cycle during growth with Fe(III) was approximately 2.4-fold larger in E1 (5 mM acetate) than E2 (H_2 _+ 1 mM acetate). This is due to the direct energy production via hydrogen oxidation which decreased the energy production demand for the TCA cycle. However, energy generation via hydrogen oxidation did not appear to completely replace energy generation via acetate oxidation by the TCA cycle. During growth in the presence of hydrogen and 1 mM acetate, flux through the TCA cycle was higher than what was required for the anabolic demand for α-ketoglutarate and oxaloacetate, and a significant amount of acetate was oxidized via the TCA cycle. One reason of the energy production via acetate oxidation in the presence of hydrogen might be the relatively poor solubility of H_2 _in water. The Henry's constant of H_2 _in water is only 7.8 × 10^-4 ^molL^-1^atm^-1 ^at 298°K and therefore the dissolved H_2 _concentration at 0.60 atm H_2 _is approximately 0.5 mM. This can also be due to other metabolic factors such as hydrogenase activity [41,42] or cell preference for generating reducing power through the oxidative TCA cycle.

In the fumarate cultures, the specific acetate uptake and TCA cycle activity was dramatically lower than that in the Fe(III) cultures (Table 3). The specific acetate uptake in the two fumarate cultures was 65 to 66 mmol acetyl-CoA per gram biomass synthesis as compared to 166 to 369 mmol of acetyl-CoA per gram biomass synthesis for the Fe(III) cultures (Table 3). The standard redox potential of the half reaction Fe^3+ ^+ *e*^- ^→ Fe^2+ ^(*E*° = 771 mV) is higher than that of fumarate^2- ^+ 2*e*^- ^+ 2H^+ ^→ succinate^2- ^(*E*° = 31 mV). Thus, it is expected that a higher electromotive force (Δ*E*°) would be generated by the acetate/Fe(III) pair than the acetate/fumarate pair. However, the fumarate cultures consumed less acetate per unit biomass formation than the Fe(III) cultures, suggesting that the cells in the fumarate cultures utilize acetate more efficiently than in the Fe(III) cultures. Higher biomass yields on acetate in the fumarate cultures relative to Fe(III) have been previously reported [2,32,36] and is likely due to the fact that Fe(III) is reduced on the outside of the cells which results in an additional energetic cost for exporting electrons.

### Fumarate Utilization

The ^13^CMFA results suggest that *G. sulfurreducens *utilizes fumarate not only as the electron acceptor for acetate oxidation but also as an additional carbon source. Based on efflux measurements by HPLC, fumarate uptake and succinate secretion were found to be very close in both fumarate cultures (*cf*. 'Steady-State Effluxes in Chemostat Cultures' section). However, the optimal solutions found by the stochastic ^13^CMFA runs resulted in larger fumarate uptake than succinate secretion (Figure 2) and the efflux estimates were within the statistical range given by the means and standard deviations determined experimentally (95% confidence interval). As a result, the small percentage (< 10%) of fumarate consumed as a carbon source in the fumarate cultures, which could not be determined by HPLC efflux measurements was discovered by ^13^CMFA with more accurate GC/MS labeling measurements.

As shown in Table 3, the uptake of fumarate increased the specific oxidative TCA fluxes of fumarase (FUM) and malate dehydrogenase (MDH) compared to that of α-ketoglutarate dehydrogenase (AKGD/OOR). Consequently, phosphoenolpyruvate carboxykinase (PEPCK/PEPCKG) was the enzyme responsible for initiating gluconeogenesis in the fumarate cultures, while pyruvate synthase (POR) initiated gluconeogenesis in the Fe(III) cultures. In the fumarate cultures, acetyl-CoA was replenished from pyruvate (PDH/POR) which was produced by phosphoenolpyruvate through 3-phosphoglycerate and serine. Between the two fumarate cultures, cells in the donor-limited condition E3 had higher net fumarate reduction, succinate secretion, oxidative TCA activities, and gluconeogenesis activities than those in the acceptor-limited condition E4 (Table 3).

**Figure 4:**
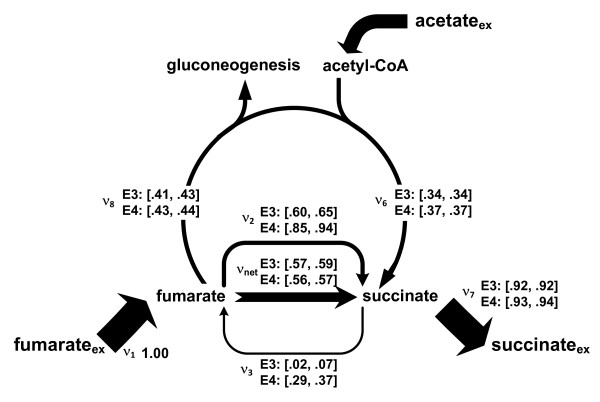
**95% confidence intervals given for key TCA fluxes (ν*_i_*) in the fumarate cultures (E3, E4), normalized by fumarate uptake**. **ν_net _**= ν_2 _- ν_3_.

To gain insight into fumarate utilization, we compared relative fluxes around fumarate and succinate, which were normalized by fumarate uptake (Figure 4). Overall, relative fumarate utilization including net fumarate reduction, fumarase flux, succinate secretion, etc., was similar in both cultures (Figure 4). A notable difference between the two fumarate cultures was found in the reversibility of FRD5. FRD5 of *G. sulfurreducens *has a dual function in fumarate reduction and succinate oxidation [32]. Both reductive (ν_2_) and oxidative (ν_3_) fluxes between fumarate and succinate were smaller in the donor-limited culture (E3) than in the acceptor-limited culture (E4). In particular, the flux through the oxidative direction was only 2 to 7% per unit fumarate uptake in E3 with the corresponding reductive flux of 60 to 65%, as compared to the oxidative flux of 29 to 37% with the reductive flux of 85 to 94% under the donor-limiting condition.

### Redox Balance

We further calculated the net production of redox equivalents (NAD(P)H and menaquinol) and carbon dioxide from the *in vivo *flux estimates. Because *G. sulfurreducens *can utilize both NADH and NADPH for Fe(III) and fumarate reduction [43,44]; NADH and NADPH are pooled for redox balance. NAD(P)H is produced in the central metabolic network by the reactions ICDHy (isocitrate → α-ketoglutarate), AKGD/OOR (α-ketoglutarate → succinyl-CoA), MDH (malate → oxaloacetate), ME1/ME2 (malate → pyruvate), PDH/POR (pyruvate → acetyl-CoA), GAPD (glyceraldehyde 3-phosphate → 1,3-bisphosphoglycerate), and ADA (acetaldehyde → acetyl-CoA). In addition, cells consume 14.2 mmol NADPH and produce 2.32 mmol NADH per gram biomass synthesis based on the genome-scale model [2]. 3-phosphoglycerate dehydrogenase involved in serine biosynthesis produces NADH, yet the next reaction carried out by 3-phosphoserine aminotransferase involves conversion of glutamate to 2-oxoglutrate for transamination. Without 2-oxoglutamate secretion, glutamate has to be regenerated from 2-oxoglutarate which consumes one redox equivalent. Hence, there is no net production of redox equivalent in this case. In *G. sulfurreducens*, FRD5 reduces menaquinone to menaquinol when succinate is oxidized to fumarate and vice versa for the fumarate reduction. The fluxes related to decarboxylation/carboxylation are ICDHy, AKGD/OOR, PC, ME1/ME2, PEPCK/PECKG, PDH/POR, and other biosynthetic pathways. In addition, cells produce about 1.36 mmol CO_2 _per gram biomass synthesis. Details of anabolic NADPH net consumption, NADH net production, and net decarboxylation are listed in additional file 1.

In the acetate/Fe(III) culture (E1), the molar yields of redox equivalents and CO_2 _net production on acetate were 3.97 ± 0.08 and 1.97 ± 0.05, respectively. These corresponded to a molar yield of Fe(III) reduction on acetate of 7.94 ± 0.16 and a ratio of Fe(II) to CO_2 _production of 4.03 ± 0.13. Comparing the stoichiometry of complete acetate oxidation with Fe(III), i.e., CH_3_COOH + 8Fe^3+ ^+ 10H_2_O → 2CO_2 _+ 8Fe^2+ ^+ 8H_3_O^+^, the Fe(III) reduction and CO_2 _production drawn from the *in vivo *flux estimates for E1 are consistent with the theoretical stoichiometry.

The direct oxidation of hydrogen coupled with Fe(III) reduction is H_2 _+ 2Fe^3+ ^+ 2H_2_O → 2Fe^2+ ^+ 2H_3_O^+ ^[33,45]. Despite hydrogen utilization, cells in the hydrogen/Fe(III) culture (E2) still produced 3.56 ± 0.05 moles of redox equivalents with 1.80 ± 0.04 moles of CO_2 _per mole of acetate taken up. These results corresponded to a molar yield of Fe(III) reduction on acetate of 7.12 ± 0.10 and a ratio of Fe(II) to CO_2 _production of 3.96 ± 0.10. Thus, Fe(III) reduction together with CO_2 _production coincides with the theoretical stoichiometry of Fe(III) reduction on acetate. In contrast to E1, cells oxidized less acetate via the TCA cycle (smaller yield of Fe(III) reduction and reduced TCA fluxes) and consequently doubled biomass yield on acetate (Figure 2 & Table 3).

The complete acetate oxidation with fumarate follows CH_3_COOH + 4C_4_H_4_O_4 _+ 2H_2_O → 2CO_2 _+ 4C_4_H_6_O_4_. The net conversion of fumarate to succinate per acetate was 2.9 ± 0.1 in the donor-limited culture (E3) and 2.5 ± 0.2 in the acceptor-limited (E4). The molar yield of CO_2 _production on acetate was comparable with the theoretical stoichiometry: it was 2.0 ± 0.5 and 1.8 ± 0.3 in E3 and E4, respectively. These results may be due to additional decarboxylation of fumarate carbons, e.g., gluconeogenesis initiated from oxaloacetate by PEPCK/PEPCKG and acetyl-CoA replenishment from pyruvate by PDH. In addition, two fumarate carbons can potentially be released through these reactions.

## Conclusions

In the present work, we characterized the intracellular metabolic response of *G. sulfurreducens *towards different electron donor/acceptor conditions *in vivo *using ^13^C-based metabolic flux analysis. The metabolic characteristics were examined from different aspects of acetate and fumarate utilization, biomass formation, and flux distributions of key metabolic branch points. Expanding on this, we compared redox balances drawn from flux estimates of the different cultures. From ^13^C labeling measurements and subsequent flux analysis, the greater metabolic discrepancies in the central metabolism, such as different initial step in gluconeogenesis, were observed to be induced by the electron acceptor variation of Fe(III) versus fumarate. As shown by the specific fluxes, metabolic activities in the TCA cycle were perturbed not only by electron acceptor variation (Fe(III) vs. fumarate) but also by electron donor variation (acetate vs. hydrogen). Furthermore, we were able to obtain detailed understanding of cellular fumarate utilization and demonstrated that fumarate acts simultaneously as the electron acceptor and an additional carbon source, as previously hypothesized elsewhere [46]. This seems to be the reason why cells in the fumarate cultures initiated gluconeogenesis via phosphoenolpyruvate decarboxykinase and resulting in the higher biomass yield on acetate than in the Fe(III) cultures.

In the hydrogen/Fe(III) culture, we found that cells still maintained the oxidative TCA cycle, with reduced activities as compared to the acetate/Fe(III) culture. From redox balance calculations, we observed that cells gained redox equivalents from both hydrogen and acetate; i.e., acetate supplemented in the hydrogen culture was not only utilized for anabolic demand but also oxidized along with hydrogen by Fe(III).

The two fumarate cultures with different ratios of acetate and fumarate gave discrepancies in metabolic fluxes per unit acetate uptake or per unit biomass synthesis including fumarate uptake and succinate secretion. Nevertheless, the two cultures appeared to have some physiological similarities. In particular, examination of TCA fluxes scaled by fumarate uptake elucidated metabolic similarities between the fumarate cultures in terms of fumarate utilization. The notable discrepancy between the two cultures was reversibility of fumarate reductase.

*G. sulfurreducens *has great potential for use in applications such as harvesting bioelectricity and environmental remediation. In these applications, understanding the metabolism of *G. sulfurreducens *with different electron donors and acceptors will allow for improved strategies or designs. Along with *in silico *studies using material and energy balances [2], the *in vivo *information gained from the ^13^C metabolic flux analysis in the present work can aid improvements of bioprocesses using this interesting microorganism. For example, the flux distributions in donor limiting or acceptor limiting conditions coupled with microbial community models may help improve bioremediation strategies. The flux results of acetate and H_2 _as donors can provide insights into certain co-cultures or microbial communities containing *G. sulfurreducens *that produce bioelectricity in microbial fuel cells, and can be used to improve designs for higher electricity production. Also, other fascinating metabolic capabilities of *Geobacter *species such as degrading variety of organic materials, including aromatic compounds [1,47] and reducing toxic and radioactive metals [45,48,49], can be investigated using ^13^C metabolic flux analysis in the future.

## Methods

### Experimental Design & Cultivation Conditions

*Geobacter sulfurreducens *(ATCC51573) [33] was cultured at 30°C under strict anaerobic conditions with an N_2_:CO_2 _atmosphere (8:20) in a defined freshwater medium [50] either in pressure tubes or in chemostats as previously described [36].

To investigate the metabolic response of *G. sulfurreducens *to variations in electron donors and acceptors, three chemostat experiments (E1, E3, E4), in which acetate was provided as the electron donor and carbon source and either Fe(III)citrate or fumarate served as the electron acceptor, were conducted. Three biological replicates were performed for each of the three experiments and chemostats were run at a dilution rate of either 0.04 h^-1 ^(E1) or 0.05 h^-1 ^(E3 and E4). Cells were harvested at steady state, after five volume refills to achieve isotopic steady-state and to make the fraction of inoculum negligible. To confirm steady-state, substrate (*s*) and biomass (*x*) concentrations were measured to verify no accumulation of substrate (*d*[*s*]/*dt *= 0) as well as biomass (*d*[*x*]/*dt *= 0).

In addition, one batch experiment (E2) was performed in which both acetate and hydrogen were provided as electron donors and Fe(III)citrate served as the electron acceptor. This experiment consisted of four replicate cultures, which were harvested and pooled when the concentration of Fe(II) approached 50 mM. All the batch cultures were transferred (1 vol/vol-%) into ^13^C-tracer containing minimal medium at least once prior to the initiation of an experiment to extremely minimize the fraction of non-labeled cells. The main cultures with the identical medium were inoculated with the mid-exponential phase ^13^C-precultures.

All cultures contained a mixture of 70% non-labeled and 30% uniformly labeled acetate (Sigma-Aldrich, St. Louis, MO) as a ^13^C-tracer carbon source for ^13^CMFA. A summary of the cultivation conditions is provided in Table 1.

### Analytical Methods

Cell growth was monitored by determining total protein by the bicinchoninic acid method with bovine serum albumin as a standard [51] and assuming a protein content of 46% of cell dry weight [2]. Organic acids were monitored by high-performance liquid chromatography (HPLC) as previously described [46].

The amino acids obtained by cell protein hydrolysis were silylated using *N*-methyl-*N*-tert-butyldimethylsilyl trifluoroacetamide as previously described [3,27]. Gas chromatography/mass spectrometry (GC/MS) analysis was carried out using a Hewlett-Packard HP G1723A GC-quadrupole mass selective detector (electron impact), equipped with a DB-5 column (Agilent Technologies). From the mass spectra of *t*-butyldimethylsilyl (TBDMS)-amino acid derivatives (triplicate measurements), carbon mass isotopomer distributions (MID) were computed using the method described earlier [27].

The metabolic fluxes were computed from the carbon mass isotopomer distributions obtained from the mass spectrometric labeling analysis of different fragment ions of TBDMS-derivatives of proteinogenic amino acid hydrolysates comprising alanine, glycine, leucine, isoleucine, serine, phenylalanine, aspartate, and glutamate (see additional file 2). The GC/MS fragment ions for those TBDMS-amino acid derivatives are listed elsewhere [19].

### ^13^C Metabolic Flux Analysis

From the carbon mass isotopomer distributions of proteinogenic amino acid hydrolysates and measured effluxes, *in vivo *metabolic fluxes were computed by solving the following constrained nonlinear least-squares minimization problem (NLSP):

(1) $$\underset{\Theta }{\min}\frac{1}{2}{\left(\eta -F(\Theta )\right)}^{T}\Sigma^{-1}\left(\eta -F(\Theta )\right)\text{ with }\Theta \in [0,1)$$

(2) $$\text{subject to }a\leq \nu \leq b\text{ \& }\nu =c(\Theta )\&\sum_{i}\nu_{carbon,i}=0$$

In the objective function (Eq. 1), the covariance-weighted (**Σ**) sum of squared of the difference between the measurements **η **(only efflux and ^13^C-labeling measurements) and the corresponding model prediction ***F***(**Θ**) are minimized with respect to [0,1)-scaled independent flux variables **Θ **(12). As the covariance, experimentally determined uncertainties associated with efflux and labeling measurements were applied. The model ***F***(**Θ**) describes the mathematical relationship between unknown fluxes (**ν**) and the measurements in terms of two connected equation systems, i.e., the stoichiometry and the carbon isotopomer reactions in the metabolic network. The fluxes **ν**, which are the dependent variables of **Θ **in the stoichiometry system, consist of immeasurable intracellular fluxes **ν**_u _and measurable effluxes **ν**_m_, i.e., **ν **= (**ν**_u_, **ν**_m_)*^T^*. Typically, realistic models are underdetermined, that is, the rank of the stoichiometric matrix **S **is smaller than the number of entries in **ν**_u_. This difference between *rank*(**S**) and the number of entries of **ν**_u _equals the number of fluxes that have to be chosen as the design parameters, **Θ**. Those independent fluxes are estimated from isotopic labeling measurements in ^13^CMFA.

In the present work, *in vivo *flux estimation was done using the elementary metabolite unit (EMU) concept [15]. To solve the NLSP (Eq. 1) with the physical constraints (Eq. 2), a gradient-based hybrid algorithm, described in detail in Yang et al. (2008) [18], was employed. The first inequality constraint in (Eq. 2) is the feasible region in the flux space allowed by the stoichiometry and experimental uncertainties given for the effluxes **ν**_m_. The region can be computed *a priori *by solving a quadratic programming problem for the stoichiometric system [2]. The second constraint is the elemental balance of carbon, i.e., the sum of carbons incoming into the system equals that of outgoing the system. Hereby, carbon dioxide is not measured but can be derived from the stoichiometry, i.e., the net CO_2 _efflux equals the difference between the decarboxylation (*ν*_decarb_) and the carboxylation (*ν*_carb_) fluxes. Hence, carbon balance can be computed from the effluxes (substrate uptake, product and biomass formation) and the net CO_2 _efflux, i.e.,

(3) $$\sum_{i=1}^{n}c_{s,i}\nu_{s,i}-\sum_{j=1}^{m}c_{p,j}\nu_{p,j}-\left(\sum_{k=1}^{p}\nu_{\text{decard},k}-\sum_{l=1}^{q}\nu_{\text{card},l}\right)=0.$$

Here, *ν*_S _and *ν*_P _denote the effluxes of substrates and products, respectively, and *c*_S _and *c*_P _the number of carbons in the corresponding compounds. From precursor relation, cells are expected to produce 1.36 mmol CO_2 _per gram dry cell weight. In practice, the constraint on carbon balance can be defined as an inequality to consider measurement uncertainties, e.g., between ± 0.05.

### Metabolic Network

The framework of ^13^CMFA is to draw up an appropriate metabolic network for the biological system of interest. Based on the genome-scale metabolic network of *G. sulfurreducens *reconstructed by Mahadevan et al. (2006) [2], the central metabolic network of *G. sulfurreducens *designed for ^13^CMFA comprises 26 intracellular fluxes containing 15 bidirectional flux pairs in the tricarboxylic acid (TCA) cycle, gluconeogenesis, pentose phosphate (PP) pathway, amino acid metabolism, and anabolic demands for biomass synthesis. The details enzyme reactions are listed in Table 2. In terms of the null space investigation of the stoichiometric matrix [52], the metabolic network was found to have 20 independent flux variables that need to be determined from ^13^C-labeling measurements.

In this metabolic network, the conversion from phosphoenolpyruvate to pyruvate is catalyzed by pyruvate kinase, whereas the reverse reaction from pyruvate to phosphoenolpyruvate by phosphoenolpyruvate synthase and/or pyruvate phosphate dikinase. Also, the conversion of pyruvate to acetyl-CoA is catalyzed by the irreversible pyruvate dehydrogenase, and the bidirectional pyruvate synthase. Enzyme reactions catalyzing the identical substrate/product pairs in the same direction cannot be discriminated by the ^13^C-based MFA.

The precursor demand of *G. sulfurreducens *applied to the present study is listed in Table 4. The anabolic fluxes from the listed precursors into biomass synthesis can be calculated by multiplying the biomass yield coefficients with the precursor demand.

### Flux Quantities

In the present work, we utilized two different flux quantities. The relative fluxes utilized in sections '*Overview of in vivo Fluxes*' and '*Fumarate Utilization*' are the rates normalized to acetate uptake and fumarate uptake, respectively. They can be understood as molar yield coefficients on a particular substrate or the rates of enzyme reactions associated with the uptake of a unit substrate in unit time.

Another flux quantity introduced in section '*Specific Metabolic Fluxes*' are the specific fluxes scaled by cell growth. We defined these specific fluxes as the relative flux *v_i _*(rate of a metabolite processed *dc_i_*/*dt *[mmolL^-1^h^-1^] per acetate uptake rate [mmolL^-1^h^-1^]) normalized by *Y_XS_*. Thus, the specific flux *q_i _*[(mmol/h) (g_biomass_/h)^-1^] of the *i*^th ^enzyme reaction equals

(4) $$\begin{array}{l} q_{i}=\frac{\nu_{i}}{Y_{XS}}=\frac{dc_{i}}{dt}{\left(\frac{dc_{X}}{dt}\right)}^{-1},\\ where\;\nu_{i}=\frac{dc_{i}}{dt}{\left(\frac{dc_{S}}{dt}\right)}^{-1}\&Y_{XS}=\frac{dc_{X}}{dt}{\left(\frac{dc_{S}}{dt}\right)}^{-1}. \end{array}$$

The specific flux can be understood as the rate of a metabolic enzyme reaction associated with the synthesis of a unit biomass in unit time.

### Software Implementation & Statistical Data Analysis

All computations involved in carbon mass isotopomer analysis and ^13^CMFA were implemented using MATLAB (Version 7.8, The Mathworks Inc., Natick, MA). Numerical optimization was carried out using the Optimization Toolbox (Version 4.2) of MATLAB.

The *in vivo *metabolic fluxes were computed stochastically by starting the numerical optimization runs from arbitrary points and by using normal random numbers of measurement data. Hereby, ^13^C labeling patterns of amino acids were computed directly if specified in the stoichiometric network model (Figure 2). Otherwise, ^13^C labeling patterns were computed from the corresponding precursors. The procedure was repeated for 500 times from different starting points to get sample distributions of the *in vivo *flux estimates resulting from the numerical optimization. The symbolic operations required for metabolic flux modeling such as parametrization and computing partial derivatives required for EMU modeling were implemented using the Symbolic Math Toolbox (Version 3.2.3) of MATLAB.

Statistical data analysis such as random number generation, confidence interval, analysis of variance (ANOVA) and multiple comparison procedure were conducted using the Statistics Toolbox (Version 7.1) of MATLAB. One-way ANOVA was implemented to test the hypothesis that the means of the flux estimates given for the different culture conditions were identical, against the general alternative that they were not. In addition to this, multiple comparisons were performed to obtain information about which pairs of flux mean estimates are significantly different or not.

### Chemicals

[U-^13^C_2_] acetate was applied to ^13^CMFA in all the four experiments (Table 1). To verify whether cells are capable of using citrate as a carbon source, [U-^13^C_6_] citrate was utilized.

## Competing interests

The authors declare that they have no competing interests.

## Authors' contributions

THY constructed the metabolic model, implemented data analysis such as GC/MS carbon labeling analysis and ^13^C metabolic flux analysis, and drafted the manuscript. MVC conceived the experimental design, conducted the experiments including analytics, and participated in the manuscript draft. DRL and JS co-supervised the work and were involved in writing the manuscript. All authors read and approved the final manuscript.

## Supplementary Material

Additional file 1 **Anabolic precursor demand**. Precursor stoichiometry given for the anabolic demand of *G. sulfurreducens *are listed. Click here for file

Additional file 2 **GC/MS mass isotopomer distributions**. Carbon mass isotopomer distributions of different amino acid fragments computed from GC/MS measurements of TBDMS-amino acid derivatives are listed. Click here for file
